
JOURNAL INFORMATION
==============================
NLM Title Abbreviation: Curr Med Sci
Journal ID: Curr Med Sci
NLM Journal ID: 101729993

Current Medical Science

ISSN: 2096-5230
EISSN: 2523-899X

ARTICLE INFORMATION
==============================
PMCID: PMC7596002
PMID: 33123913
DOI: 10.1007/s11596-020-2256-3
Article ID: 2256
Article version: 1
Subjects: Erratum

Erratum to: Changes of Serum Insulin-like Growth Factor-2 Response to Negative Symptom Improvements in Schizophrenia Patients Treated with Atypical Antipsychotics

Chao Xue-lin 1
Jiang Shu-zhen 2
Xiong Jian-wen 3
Zhan Jin-qiong 2
Wei Bo 2 3
Chen Chun-nuan 4
Yang Yuan-jian 2 3
1 grid.412604.5 0000 0004 1758 4073 Department of Psychosomatic Medicine, The First Affiliated Hospital of Nanchang University, Nanchang, 330006 China
2 grid.260463.5 0000 0001 2182 8825 Biological Psychiatry Laboratory, Jiangxi Mental Hospital, Affiliated Mental Hospital of Nanchang University, Nanchang, 330029 China
3 grid.260463.5 0000 0001 2182 8825 Department of Psychiatry, Jiangxi Mental Hospital, Affiliated Mental Hospital of Nanchang University, Nanchang, 330029 China
4 grid.256112.3 0000 0004 1797 9307 Department of Neurology, The Second Clinical Medical College, The Second Affiliated Hospital, Fujian Medical University, Quanzhou, 362000 China
Electronic publication date: 2020 Oct 29
Print publication date: 2020
Volume: 40
Issue: 5
First page: 997
Last page: 997
Copyright: © The Author(s) 2020
License: Open Access This article is licensed under a Creative Commons Attribution 4.0 International License, which permits use, sharing, adaptation, distribution and reproduction in any medium or format, as long as you give appropriate credit to the original author(s) and the source, provide a link to the Creative Commons licence, and indicate if changes were made.
The images or other third party material in this article are included in the article’s Creative Commons licence, unless indicated otherwise in a credit line to the material. If material is not included in the article’s Creative Commons licence and your intended use is not permitted by statutory regulation or exceeds the permitted use, you will need to obtain permission directly from the copyright holder.
To view a copy of this licence, visit http://creativecommons.org/licenses/by/4.0/.
License URL: https://creativecommons.org/licenses/by/4.0/

Erratum for: Changes of Serum Insulin-like Growth Factor-2 Response to Negative Symptom Improvements in Schizophrenia Patients Treated with Atypical Antipsychotics. 40 3 17 7 2020 563 569 Curr Med Sci 10.1007/s11596-020-2214-0 32681260
issue-copyright-statement: © Huazhong University of Science and Technology 2020

==============================
The original article can be found online at 10.1007/s11596-020-2214-0

